# Nanotechnological approaches for efficient N2B delivery: from small-molecule drugs to biopharmaceuticals

**DOI:** 10.3762/bjnano.15.113

**Published:** 2024-11-12

**Authors:** Selin Akpinar Adscheid, Akif Emre Türeli, Nazende Günday-Türeli, Marc Schneider

**Affiliations:** 1 MyBiotech GmbH; Industriestraße 1B, 66802 Überherrn, Germany; 2 Department of Pharmacy, Biopharmaceutics and Pharmaceutical Technology, PharmaScienceHub, Saarland University, Campus C4 1, Saarbrücken D-66123, Germany

**Keywords:** antibody delivery, biopharmaceutical delivery, blood–brain barrier (BBB), CNS diseases, drug delivery, hybrid nanoparticles, intranasal delivery, liposomes, nanomedicine, nanostructured lipid carriers (NLCs), polymer nanoparticles, RNA delivery, solid lipid nanoparticles (SLNs)

## Abstract

Central nervous system diseases negatively affect patients and society. Providing successful noninvasive treatments for these diseases is challenging because of the presence of the blood–brain barrier. While protecting the brain’s homeostasis, the barrier limits the passage of almost all large-molecule drugs and most small-molecule drugs. A noninvasive method, nose-to-brain delivery (N2B delivery) has been proposed to overcome this challenge. By exploiting the direct anatomical interaction between the nose and the brain, the drugs can reach the target, the brain. Moreover, the drugs can be encapsulated into various drug delivery systems to enhance physicochemical characteristics and targeting success. Many preclinical data show that this strategy can effectively deliver biopharmaceuticals to the brain. Therefore, this review focuses on N2B delivery while giving examples of different drug delivery systems suitable for the applications. In addition, we emphasize the importance of the effective delivery of monoclonal antibodies and RNA and stress the recent literature tackling this challenge. While giving examples of nanotechnological approaches for the effective delivery of small or large molecules from the current literature, we highlight the preclinical studies and their results to prove the strategies’ success and limitations.

## Introduction

The central nervous system (CNS) consists of the brain and the spinal cord and is considered the body’s processing and control center. While the brain controls most of the functions in the body, the spinal cord carries messages from the brain to the other parts of the body [1]. Like other systems and parts of the human body, the CNS is susceptible to various disorders [2]. CNS diseases are a group of challenging pathological conditions such as multiple sclerosis, Alzheimer’s disease, and Parkinson’s disease [3]. This group of diseases affects both patients and society and causes one in nine deaths worldwide [4]. For example, solely in 2019, there were nearly 10 million deaths, and 349 million disability-adjusted life years were estimated due to neurological diseases [5]. The estimated cost of these diseases to the European healthcare budget is around 800 billion Euros per year [6].

**Figure 1:**
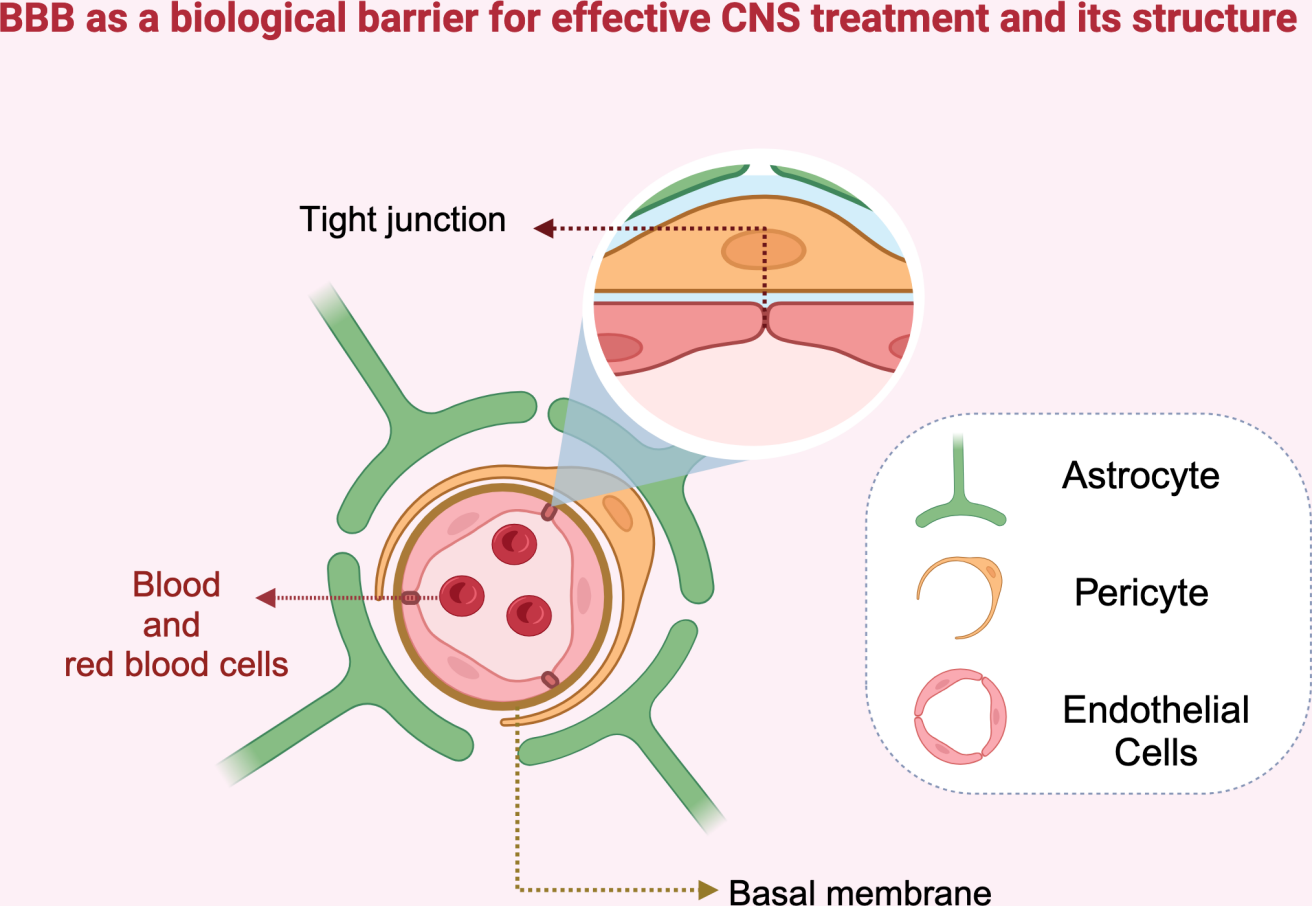
Structure of the blood–brain barrier. The endothelial cells are joined by tight junctions to form a barrier for metabolic functions. The barrier is surrounded by a basal membrane, pericytes, and astrocytes. Figure 1 was redrawn from [14] and created in BioRender. Akpinar, S. (2023) https://BioRender.com/c51s574. This content is not subject to CC BY 4.0.

From a pharmaceutical point of view, although there is an immense network of cerebral vascularization, providing therapeutics for CNS diseases is considered a challenge because of the existence of the blood–brain barrier (BBB, Figure 1), which is composed of several cell types [7]. The BBB is a dynamic and selective interface between the systemic circulation and the brain [8]. The structure of the healthy BBB relies on the endothelial cells and the tight barrier formed using tight junctions [9–11]. These are surrounded by other cell types, such as astrocytes and pericytes. Astrocytes are crucial for the formation and maintenance of the BBB, which leads to an adequate association between the cells and the BBB. Pericytes are also important regulatory cells for the homeostasis of the BBB. The interaction between astrocytes and pericytes plays a vital role in brain vasculogenesis and the maintenance of the BBB [12]. Overall, the high selectivity of the BBB provides optimal conditions for CNS homeostasis [13].

Because of the presence of the BBB, most systemically administered drugs cannot reach the brain as a site of action. The BBB is reported to limit the passage of 98% of small-molecule drugs and almost all large-molecule therapeutics to the brain [15]. The high density of the intact barrier prevents an easy penetration of the barrier. The techniques to overcome the BBB can be invasive and noninvasive. As part of the invasive methods, disruption of the BBB with osmotic pressure and intrathecal delivery have been proposed [16]. As examples of noninvasive methods, intranasal drug delivery and bypassing the BBB by nanoparticles can be counted. While the systemic route of administration with the combination of drug delivery systems (DDSs) to cross the BBB has been promising, the efficiency is often not yet satisfactory [17]. Another noninvasive technique, nose-to-brain delivery or nasal-to-brain delivery (N2B delivery), in contrast, bypasses the BBB through a direct connection [18–19]. When combined with nanotechnology, N2B delivery becomes even more attractive since nanotechnology-driven DDSs have enabled improved drug accumulation at the target site [20].

DDSs, such as polymeric or lipid-based nanoparticles (NPs), can provide an opportunity to modify the release profile of the drugs, enhance targeting efficiency, and improve nasal permeation during intranasal administration [21–24]. In general, the encapsulation of active pharmaceutical ingredients (APIs) into mucoadhesive DDSs can mitigate rapid mucociliary clearance [25–26] and protect the drug from biological or chemical degradation or deactivation, which results in increased bioavailability in the brain [18,27]. This is particularly important since many other routes of administration pose multiple barriers and lead to relevant drug degradation, which are huge challenges for drug delivery. In intranasal administration, the structure of the nose allows for an anatomical option to overcome some of those barriers. Looking at N2B, one of the most significant barriers is the nasal mucosa, where the drugs can be rapidly cleared through mucociliary clearance. While it is a significant challenge for delivery, the same effect is not as significant in the olfactory region, making this region one of the targets of the N2B delivery. However, in this case, the limited surface area of the region is considered a challenge to efficient administration [28]. This route of administration also suffers from enzymatic degradation including peptidase and protease activity, making it challenging to deliver peptides and proteins [29–30]. Yet, the intranasal route still yields lower enzymatic degradation and higher bioavailability in the brain [31]. While the challenges of the administration route and the barriers are clear, understanding the nasal anatomy and barriers as well as addressing different efficient formulations with DDSs for N2B delivery applications remain open issues.

In recent years, biopharmaceuticals been shown to have great potential as therapeutics [32]. However, their passage to the brain is limited by the BBB, and they present incompatibilities with the oral route of administration because of their tendency to degrade [33–34]. In fact, this limitation is more pronounced for large molecules than for small molecules. As an alternative, researchers focus on intranasal administration of biopharmaceuticals to target the brain. In addition, using DDSs to deliver biopharmaceuticals to the CNS can present additional advantages such as enhanced retention on the mucosal surface, drug stability, and bioavailability [35].

In this review, we focus on the recent developments in N2B delivery and discuss the structure of the nasal anatomy and the principles of intranasal administration, the principles of the DDSs for N2B delivery, and the N2B delivery of biopharmaceuticals. To provide a current overview on the studies conducted in this field, we focus on work published in the last five years (2019–2024). We also enriched the review with different studies from the last decade to highlight important previous scholarly work.

## Review

### Nasal anatomy and intranasal drug delivery: advantages and limitations

**Figure 2:**
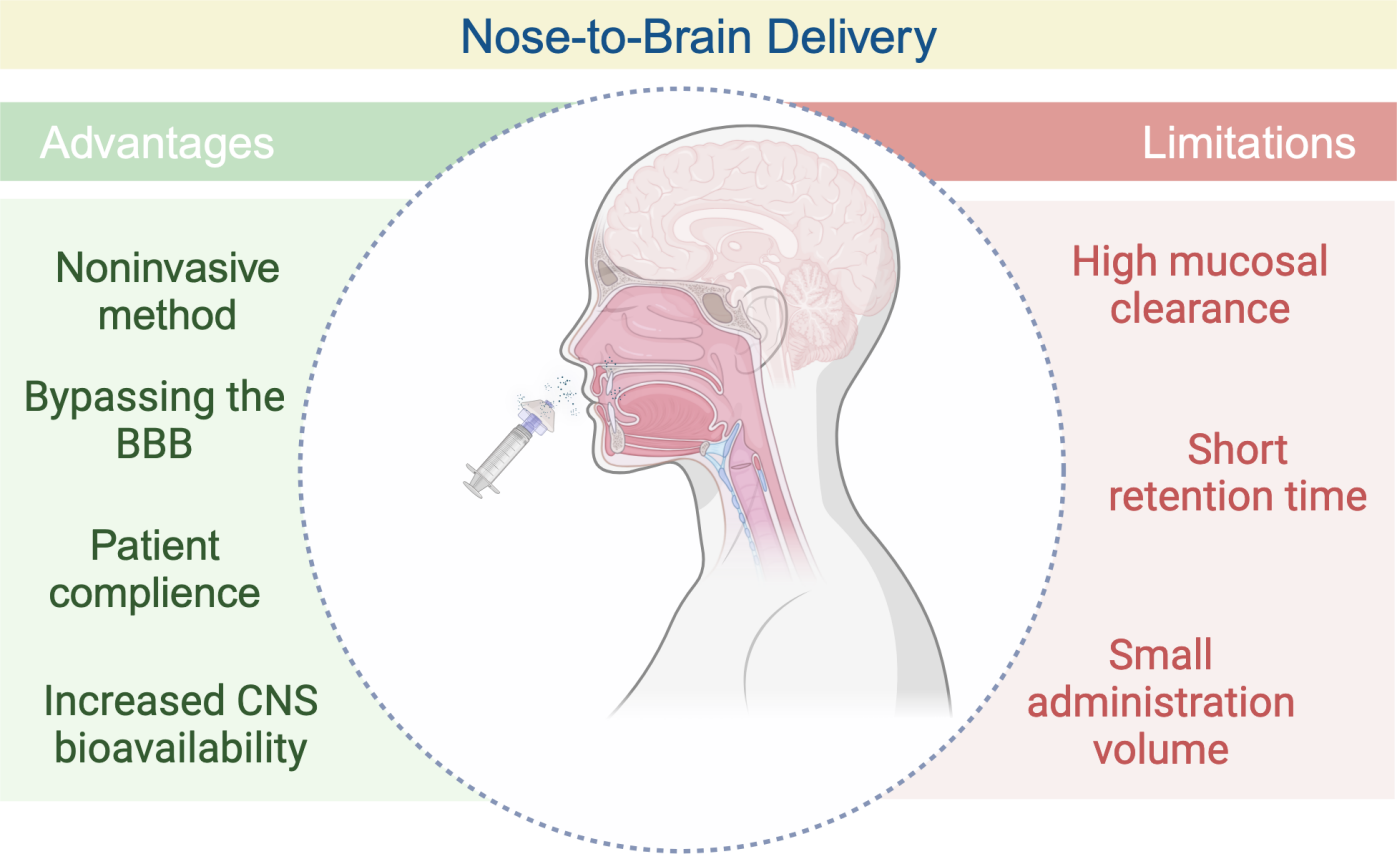
Advantages and limitations of N2B delivery. Figure 2 was created in BioRender. Akpinar, S. (2023) https://BioRender.com/t58u758. This content is not subject to CC BY 4.0.

The nasal route of drug administration has gained attention over the last decade and has become more attractive, especially with the COVID-19 pandemic vaccine development research [36]. The anatomy of the nose allows for noninvasive administration and plays a critical role in intranasal delivery [37]. The nasal cavity has a highly vascularized anatomy and offers a relatively large surface area [38]. Compared to the many other routes of administration, it also has a more permeable structure [39]. These advantages make the nasal route suitable for local delivery and systemic administration [40]. It offers various benefits, including but not limited to a fast onset of action [41]. Intranasal delivery can be used to target a limitedly available site, the brain [42]. These N2B delivery applications are based on the olfactory region of the nasal cavity. Such N2B delivery applications exploit the direct anatomical connection between the brain and the olfactory region [43]. These direct anatomical interactions make the olfactory region the primary target for N2B delivery and bypass the BBB [21,44]. However, some factors, such as high mucociliary clearance and short retention time, small dosage volume, and the need for a drug delivery device, limit N2B delivery (Figure 2) [45–46].

### Anatomy of the nasal cavity: a brief introduction

The nose has a complex anatomical structure and is responsible for the olfactory and respiratory actions of the human body. The nasal cavity comprises the vestibule, as well as olfactory and respiratory regions [47]. The nasal vestibule is the anterior part of the nasal cavity. The latter parts, olfactory and respiratory regions, have the possibility for further drug absorption but differ in their routes of drug delivery pathways. Although the olfactory mucosa is predominantly targeted for N2B delivery applications, the respiratory mucosa is also being investigated because it is innervated by the trigeminal nerve and provides a possible indirect transport of the drugs to the brain via a systemic pathway [48].

The focus of research interest is the olfactory region in the upper part of the nasal cavity lined up with the olfactory epithelium. It consists of three primary parts, namely, olfactory epithelium, lamina propria, and the basement membrane [49]. The olfactory epithelium is the specialized epithelial tissue mainly formed by basal, supporting, and olfactory sensory neurons [50]. The olfactory sensory neurons are bipolar neurons and chemoreceptors, which play an essential role in recognizing odor [51]. They deliver information to the olfactory bulb, where it is processed [52]. Below the olfactory epithelium is another part of the olfactory region, the lamina propria, which contains different cell types, including Bowman’s glands, responsible for producing mucus [53]. The lamina propria is also the region where the olfactory sensory neurons start and form the installments of the olfactory sensory neurons [54]. The respiratory mucosa, in contrast, lines with the respiratory epithelium and contains mainly ciliated cells, goblet cells, and basal cells [55].

### Transport mechanisms for N2B delivery

**Figure 3:**
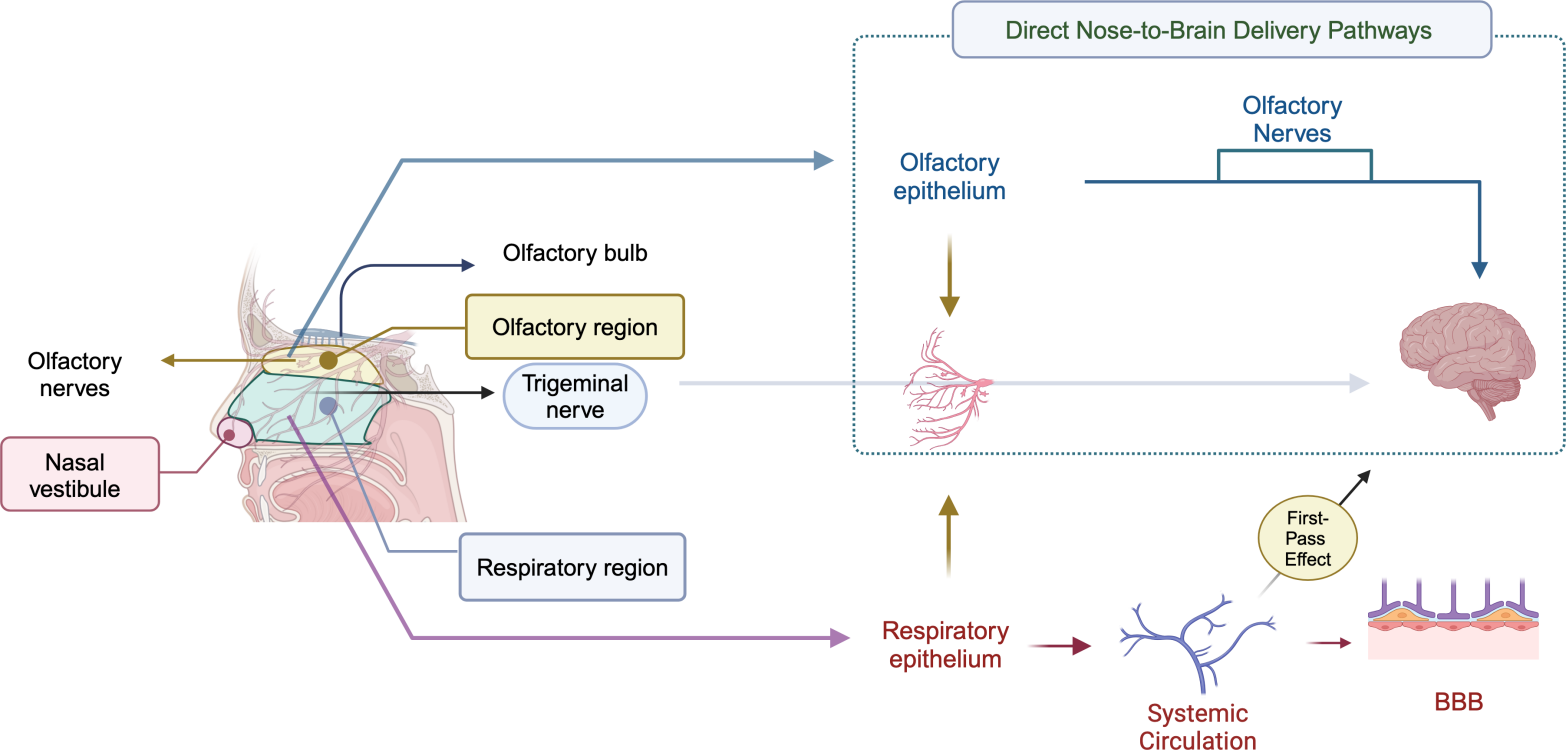
The anatomy of the nasal cavity and the suggested direct and indirect transport mechanisms for N2B delivery. The direct transport of the drugs can be within the olfactory epithelium or the trigeminal nerve. Figure 3 was redrawn from [59] as well as [60] and created in BioRender. Akpinar, S. (2023) https://BioRender.com/h18x614. This content is not subject to CC BY 4.0.

The transport mechanisms for the N2B delivery are yet to be fully understood. It is suggested that olfactory and trigeminal nerves are responsible for direct transport to the brain through the nasal route (Figure 3). For the olfactory pathway, the drugs pass through the cribriform plate and are projected to the olfactory bulb [21]. In addition to the olfactory path, there is the trigeminal pathway, the largest and fifth cranial nerve [56]. Even though the trigeminal pathway is less explored than the olfactory pathway, a study by Li et al. showed that the trigeminal pathway could be the dominant pathway for the N2B delivery of intact polymeric NPs [57]. Furthermore, because of the highly vascularized nature of the respiratory region of the nose, drug transport can also follow a systemic pathway through this region [58].

N2B drug transport includes intracellular, paracellular, and extracellular mechanisms [42]. While the intracellular pathway is based on the drug’s internalization by the neurons followed by axonal transport, the extracellular pathway is based on the drugs’ transport through the paracellular space [61–62].

### Drug delivery systems for N2B delivery applications

**Figure 4:**
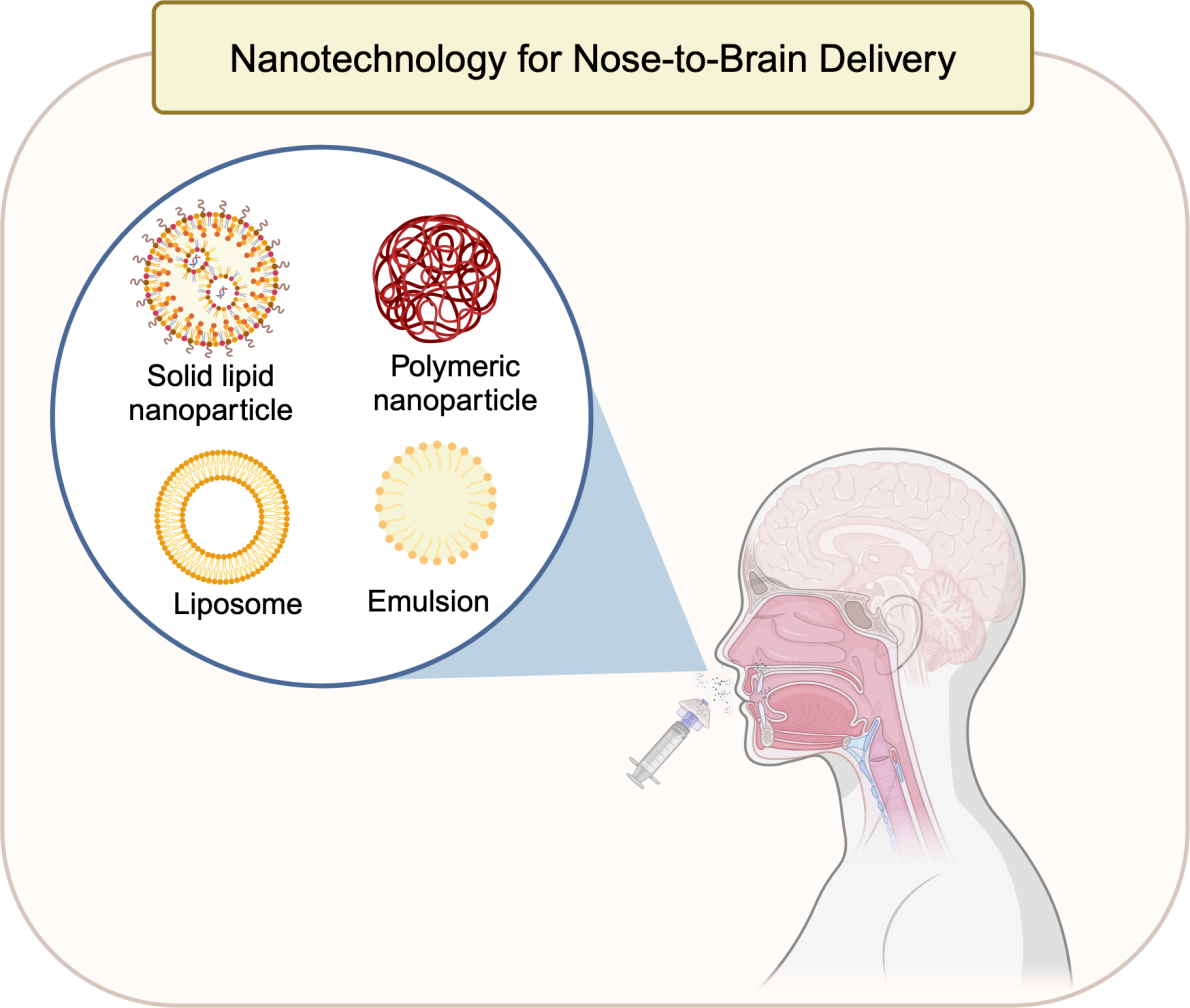
Different potential nanocarriers for N2B delivery: SLNs, polymeric NPs, liposomes, and emulsions. Figure 4 was created in BioRender. Akpinar, S. (2023) https://BioRender.com/r14d536.

DDS can deliver the encapsulated APIs to the brain using olfactory or trigeminal pathways while protecting the drug from biological and chemical degradation. Moreover, they can provide target-specific delivery via surface modifications and provide significant tailorable release properties to the encapsulated drugs. N2B delivery applications present increased efficacy and safety of the drugs in contrast to application of free drugs [63]. The physicochemical characteristics of the DDSs are important in determining the success of drug carriers for CNS targeting. For example, size, shape, and surface characteristics of a DDS directly affect cellular transport and uptake, biodistribution, and the interaction with biological interfaces [64–65]. Regarding particle size, NPs with a size of approx. 15 nm or below were observed to penetrate the olfactory bulb thanks to the paracellular space in the olfactory epithelium [66]. However, because of their small particle size, they could be more suitable for imaging applications rather than drug delivery. Moreover, NPs with sizes up to 300 nm were found and considered suitable for intranasal delivery [67–68]. Significant portions of the NPs studied for N2B delivery are approx. 200 nm, which is the average size of olfactory exons [44,60]. Rejman et al. demonstrated that the clathrin-mediated pathway of endocytosis has an upper limit for internalization of approximately 200 nm. Their study also revealed that an increase in particle size led to a shift towards caveolae-mediated internalization, the primary pathway for particles sized around 500 nm [69]. This shows the possibility of an even more extended particle size range to be considered for N2B delivery [69]. Regarding surface characteristics, chitosan-coated NPs can be a good example, as positively charged chitosan and its derivatives interact with negatively charged mucin. This interaction can enhance the NPs’ residence time in the nasal cavity [70]. To delve into different properties of these DDSs in detail, the next section focuses on solid lipid NPs, polymeric NPs, liposomes, emulsions, and novel hybrid NPs and their potential use as DDSs in N2B delivery (Figure 4).

### Polymeric NPs

Because of their tunable physicochemical characteristics, polymeric NPs are a potential vehicle for different drug delivery applications [71]. They can be prepared with simple production methods [72], and there are also efforts made regarding reasonable upscaling [73–76]. Among the natural polymers, chitosan and its derivatives could provide many advantages to brain delivery because of their mucoadhesive properties, which increase mucosal retention [77]. In addition to biopolymers, synthetic biodegradable and biocompatible polymers such as poly(lactic acid) (PLA) and poly(lactic-*co*-glycolic acid) (PLGA), which are approved by the US Food and Drug Administration (FDA) for human administration [78] are relevant options.

Extensive testing has been conducted for polymeric nanoparticles to understand their role in N2B delivery. For instance, Gabold et al. reported the preparation of protein-loaded chitosan NPs decorated with transferrin as a proof-of-concept study to demonstrate a versatile surface functionalization that can also be suitable for N2B delivery. They showed that the transferrin-decorated NPs with the highest amount of targeting ligand exhibited the highest cellular uptake in the RPMI 2650 human epithelium cell line [79].

In another study, the researchers studied the chitosan coating of PLGA NPs regarding the mucosal uptake. Chatzitaki et al. reported the encapsulation of ropinirole hydrochloride (RH), an anti-Parkinson drug, into chitosan-coated PLGA NPs by the nanoprecipitation method to enhance drug delivery through the nasal mucosa. The researchers assessed the mucoadhesive properties by observing the ability of the NPs to adsorb mucin. While the presence of the mucin did not significantly alter the negative surface charge of the PLGA NPs, the more negative zeta potential values of the PLGA-chitosan NPs showed that there was an interaction with mucin. Following this, the RH-loaded NPs showed 3.22-fold enhanced drug permeation through sheep nasal mucosa compared to their non-coated PLGA counterparts [80].

In another study by Spindler et al., the researchers prepared PLGA NPs in batches of different sizes, as well as chitosan-coated PLGA NPs, to study the uptake mechanisms of these particles into the olfactory mucosa through ex vivo permeation studies. The PLGA NPs showed size- and time-dependent uptake mechanisms. For instance, PLGA NPs with particle sizes of 80 and 175 nm were taken up after only 5 min in the lamina propria; after 15 min, PLGA NPs with a particle size of 520 nm were associated with nuclei. Additionally, the authors presented that PLGA NPs with larger particle sizes (520 nm) were accumulated in neural bundles. They showed that this accumulation could enable intracellular uptake in neuronal axons and further provide transcellular transport with the olfactory or trigeminal nerve pathways [81]. It should be noted that while ex vivo studies provide advantages such as simplicity of the procedures and high tissue availability, differences in tissue morphologies should be considered [82]. These results regarding the larger particle size (520 nm) are particularly interesting.

Although particle sizes generally considered suitable and reported for N2B delivery are 200 nm and below [83], a computational study revealed that NPs with a size of 500 nm could also be deposited in the olfactory region (although to a lesser extent than the smaller NPs); however, it should be noted that the computational model only provides limited insights as some factors, such as injection position and the effect of the mucus layer, were not considered [84]. The study also highlighted that the chitosan coating favored the penetration in the mucosa. This was attributed to the swelling of chitosan at the pH value of the healthy nose and the well-studied tight junction opening abilities of chitosan [85].

In another study on the mucoadhesion and permeation properties of materials, a thiolated cellulose was synthesized and used for delivering a model drug, enoxaparin. The authors reported that thiolation increased mucoadhesion by a factor of 9.6 in porcine intestinal mucosa, while the apparent permeability was increased 2.2-fold. These results showed that thiolated cellulose could also be a candidate for enhancing mucoadhesion and permeability, and this polymer could also be studied regarding nasal mucosa permeation in the future [86].

### Liposomes

Liposomes are another type of DDS that has been extensively investigated over the years [87–89]. The structure of a liposome contains a lipid bilayer surrounding an aqueous core, which offers advantages in encapsulating both hydrophobic and hydrophilic substances [90]. The additional advantages of liposomes include, but are not limited to, high biocompatibility, biodegradability, and prolonged retention in the blood stream when pegylated [91]. Despite their significant advantages, liposomes could also suffer from disadvantages such as short half-life in the body environment and drug leakage [92]. However, liposomes are still considered one of the most prominent DDS classes and have seen significant commercialization with twenty-one approved products [93]. While liposomal formulations have been on the market for some time, no liposomal product for CNS diseases exists. Even though they can act as lipophilic DDSs, because of their size, they generally cannot cross directly the BBB and are instead transported via adsorptive-mediated transcytosis and receptor-mediated transcytosis [94–95]. The literature addresses different examples of liposomal formulations for N2B delivery as an alternative route of administration [60]. For instance, Dhaliwal et al. presented a cationic liposomal formulation loaded with luciferase mRNA to evaluate the intranasal delivery to the brain in a murine model. Positively charged liposomes both enhance the interactions with anionic mRNA, leading to complexes between these materials, and the intracellular uptake for gene delivery. The authors encapsulated luciferase mRNA in the cationic liposomal formulation to quantify the mRNA expression distribution in the brain. The results of the in vivo studies with mice showed a dose-dependent increase in luciferase activity in the whole brain after the administration. Moreover, compared to the mRNA administration without liposomal formulation, the encapsulated mRNA showed higher mRNA expression than the control group, indicating successful delivery of mRNA through intranasal delivery [96]. While the results are promising, it should be also noted that most of the in vivo studies are conducted with mice and rats. However, nasal administration depends on multiple factors from administration device to the total volume of administration, and these are limited for small animals such as mice and rats. In fact, rats were found to be very different from humans. Large animals (e.g., rabbits and dogs) are more suitable for assessing in vivo pharmacokinetics and pharmacodynamics more accurately and as closely as possible to the human situation [24].

In another study by Salade et al., the researchers designed chitosan-coated ghrelin-loaded liposomal formulations to be introduced intranasally as an alternative to exogenous ghrelin administration to manage cachexia. The results highlighted different advantages of anionic liposomes over cationic liposomes. Anionic liposomes protected the drug from enzymatic degradation and showed higher encapsulation efficiency for the positively charged ghrelin at pH 7.4, indicating that the choice of the anionic/cationic liposomes should be based on the desired application as well as the encapsulated substance. Moreover, a chitosan chloride coating increased mucin adsorption by approximately 30% compared to non-coated counterparts, where this derivative was chosen because of its solubility at physiological nasal pH [97]. Additionally, the powder form of the formulation showed stronger mucin adhesion and better enzymatic degradation protection [98]. Moreover, researchers developed in situ esculin-loaded nanoliposomes for intranasal administration for treating Parkinson’s disease. Ex vivo studies further confirmed the enhanced permeation of the nanoliposomal formulation compared to the suspension form by approximately 40% [99].

### Solid lipid NPs and nanostructured lipid carriers

Solid lipid NPs (SLNs) are prepared from lipids that are solid at room temperature, which are then stabilized by surfactants [100]. While both liposomes and SLNs are considered biocompatible and biodegradable, SLNs provide increased stability because of their rigid core [101]. Nevertheless, SLNs suffer from the low drug loading capacity and drug expulsion during storage because of an increased crystallization tendency [102]. Researchers developed a second generation of lipid-based drug carriers called nanostructured lipid carriers (NLCs). Unlike the SLNs, the core structure of the NLCs is composed of both liquid and solid lipids. The non-ideal crystalline structure of the NLCs reduces the crystallinity degree, thus offering enhanced drug loading capacity while preventing drug expulsion [103]. Because of their advantages, such as improved encapsulation of lipophilic drugs and increased brain availability, SLNs and NLCs have been previously studied for delivery through the BBB [104–105].

SLNs and NLCs were also in focus for the development of intranasal formulations. For instance, rotigotine, a drug with low oral bioavailability, was loaded in SLNs to be tested against Parkinson’s disease. The optimized formulations demonstrated long stability and permeability through goat nasal mucosa [106]. Zafar et al. also encapsulated rotigotine in chitosan-coated NLCs. They optimized the chitosan-coated NLCs by design of experiment (DoE) using a three-level Box–Behnken design, and the final formulation presented a mean particle size of 170 nm, a PDI value smaller than 0.2, and a mean entrapment efficiency of approximately 82%. A significantly higher permeation of goat nasal mucosa was shown for the optimized formulation as well as an enhanced bioavailability (3.2-fold higher bioavailability for the optimized formulation compared to the intranasal application of API suspension and a 2.1-fold higher bioavailability compared to the intravenous application of the same formulation) [107]. In another study on chitosan-coated NLCs, Yasir et al. encapsulated donepezil, an anti-Alzheimer’s drug, for N2B delivery. The optimized formulation showed a mean particle size of 192 nm with good entrapment efficiency close to 90%. The study also presented a higher bioavailability for the NP formulation compared to the drug in solution (by a factor of 2.02), as well as higher bioavailability in the brain for the intranasal administration compared to the intravenous administration of the NP formulation (by a factor of 2.41) [108]. In another study, NLCs were designed and optimized by 3^2^ full factorial design for the encapsulation of clozapine, an anti-schizophrenia drug suffering from low bioavailability after oral administration. The optimization process yielded optimized NLCs with a particle size of 178 nm of and an entrapment efficiency of 77%. The in vivo pharmacokinetic study showed that intranasal administration of the optimized NLC formulation led to significantly faster drug absorption and greater clozapine concentration in the brain by a factor of 6.15 compared to the oral clozapine tablet [109].

### Nanoemulsions

Emulsions are colloidal systems consisting of two immiscible liquid phases where one phase is dispersed in the other. Nanoemulsions can be prepared via low-energy or high-energy methods, including microfluidics and high-shear-force homogenization [110]. As DDS, nanoemulsions can be reservoirs for encapsulating hydrophobic substances [111]. Moreover, emulsions of emulsions or double emulsions can be prepared by dispersing the droplets of primary emulsion into another liquid phase. Double emulsions can be a reservoir for hydrophilic substances. They decrease bioactive degradation and are also used as DDSs for the nasal administration route [102]. As an example of nanoemulsions for intranasal drug delivery, Gaba et al. developed vitamin E/naringenin nanoemulsions to treat Parkinson’s disease. The in vivo studies with Wistar rats showed that the concentration of the drug in the brain was significantly higher (approx. 3.34-fold) when the drug was encapsulated into nanoemulsions and when the formulation was administrated via the intranasal route instead of the intravenous route This result presents an even higher drug concentration compared to previously presented differences in drug bioavailability in the brain after intravenous and intranasal administration using NLCs by Patel et al. and Zafar and colleagues. This could be attributed to the formulation development and the small globule size (approx. 38 nm) enhancing the transport of the drug via the nasal route compared to the other formulations with particle sizes larger than 170 nm. While NPs with particle sizes smaller than 200 nm are generally considered optimal for N2B delivery studies, NPs with significantly smaller particle sizes, such as above case (38 nm), could also enhance the transcellular pathway through olfactory neurons and could provide advantages in N2B delivery [112].

Choudhury et al. encapsulated rotigotine into mucoadhesive nanoemulsions to test the effect of mucoadhesive properties in ex vivo permeation studies. Although more relevant conclusions can be made from in vivo studies as they also represent different barriers and the complexity of living organisms, the simplicity of ex vivo studies is advantageous as they focus directly on the permeation process providing straightforward data that can be used before, and complimentary to, in vivo studies [82]. In the ex vivo permeation studies, the mucoadhesive nanoemulsions demonstrated a 1.40-fold enhancement in permeability through nasal mucosa compared to non-mucoadhesive nanoemulsions [113]. Recently, mucoadhesive nanoemulsions loaded with mefenamic acid and stabilized with tocopherol polyethylene glycol succinate (TPGS) were proposed against Alzheimer’s disease. TPGS, a water-soluble precursor of vitamin E, was used in the formulation to reduce the amyloid-beta-induced oxidative stress [114]*.* In vivo tests on Wistar rats highlighted that the mucoadhesive nanoemulsion formulation with a particle size of 91.20 nm increased the drug absorption into the brain via the intranasal route compared to the mucoadhesive suspension form [115]. The encapsulation of a poorly soluble drug, cannabidiol, into nanoemulsions for the treatment of epilepsy was also evaluated. The intranasal administration of the formulation also showed better permeation in ex vivo studies with goat nasal mucosa and higher drug concentration in the brain in in vivo studies with Wistar rats compared to plain cannabidiol [116].

### Hybrid nanoparticles

Each NP class represents exclusive advantages for therapeutic applications. For example, polymeric NPs exhibit easily tunable surface properties [117], lipid NPs offer high bioavailability and -compatibility [118], and inorganic NPs provide unique magnetic or optical properties [119]. Moreover, DDSs can be conjugated with ligands and coated with mucoadhesive materials to tailor them for therapeutic applications. Because of the unique benefits and to avoid limitations such as burst release, low encapsulation efficiency, and toxicity [120], researchers have been combining NPs to develop hybrid NPs. Hybrid NPs are nanoparticles prepared by a combination of at least two NP classes to enhance the applications of the DDSs, which exhibit unique properties that are not possible by using only one single NP class [121]. For instance, there has been a growing interest in developing lipid–polymer hybrid NPs to benefit from the properties of different NP classes. Lipid–polymer hybrid NPs are core–shell structures with a polymeric core and lipidic coat [122]. While drug loading and permeability are regulated by the lipidic coat, the polymeric core determines the release properties of the NPs [123]. Lecithin (lipid)–chitosan (polymer) NPs have been studied for N2B delivery for poorly soluble drugs [124]. In addition to lipid–polymer hybrid NPs, inorganic–polymer and organic–polymer NPs have also been studied. In Table 1, we present different hybrid NPs developed in the past couple of years for N2B delivery, and we describe key findings from ex vivo and in vivo studies in Table 2.

**Table 1:** Summary of hybrid NPs for N2B delivery.

Hybrid NPs	Drug	Disease	Particle size	Zeta potential	Ref.

lipid–polymer	rivastigmine–DHA ion-pair complex	Alzheimer’s disease	160.8 ± 0.5 nm (negatively charged NPs); 132.4 ± 3.8 nm (positively charged NPs)	−39.3 ± 1.2 mV (negatively charged NPs); +36.4 ± 0.6 mV (positively charged NPs)	[125]
	selegiline	Parkinson’s disease	184.8 ± 72.5 nm to 226.7 ± 82.1 nm	−16.23 ± 1.3 mV to −28.21 ± 2.4 mV	[126]
	ergotamine & caffeine	migraine	239.5 ± 2.3 nm	−18.4 ± 6.6 mV	[127]
gold–iron oxide	therapeutic miRNAs	glioblastoma	53.2 nm	3.8 mV	[128]
carbon–polymer	galantamine	Alzheimer’s disease	241.6 ± 0.3 nm	−19.8 ± 0.1 mV	[129]
lecithin–chitosan	rotigotine	Parkinson’s disease	108.0 ± 4.0 nm	+14.9 ± 0.5 mV	[130]
	pripedil	Parkinson’s disease	147.5 ± 7.9 nm	+18.1 ± 0.6 mV	[131]
	simvastatin	neuroinflammatory diseases	217.8 ± 12.1 nm	+44.3 ± 2.1 mV	[132]
	phenytoin	epilepsy	120.4 ± 38.4 nm (optimal NP)	52.2 ± 0.5 mV (optimal NPs)	[124]
polymer/surfactant/cyclodextrin	ropinirole hydrochloride	Parkinson’s disease	various	from −20.5 ± 6.0 mV to −6.1 ± 2.0 mV	[133]

**Table 2:** Summary of hybrid NPs for N2B delivery: key features and significant results.

Hybrid NPs	Key features	Significant ex vivo or/and in vivo results	Ref.

lipid–polymer	the ion-pair complex enhanced the drug loading, and the cationic NPs showed enhanced amyloid inhibition	the thermoresponsive gel is embedded in hybrid NPs; enhanced pharmacokinetic parameters compared to drug–gel formulation	[125]
	NPs were prepared by the single emulsion method and showed a high entrapment efficiency of more than 80%	enhanced brain availability in comparison to an orally applied solution	[126]
	the NPs with lipid/polymer ratio of 15% showed controlled release profile and a high entrapment efficiency of >86%	4.35-fold enhanced brain uptake via intranasal administration	[127]
gold–iron oxide	NPs functionalized with chitosan–cyclodextrin enabled negatively charged miRNAs and provided a suitable particle size for intranasal delivery	when mice were treated with hybrid NPs (T7-polyGIONs loaded with miR100/antimiR-2), they had increased survival compared to control groups	[128]
carbon–polymer	absorption of the drug to the hierarchical porous carbon positively influenced the release profile of the drug	reached the hippocampus only one hour after administration	[129]
lecithin–chitosan	increased mucociliary transport time was attributed to the presence of chitosan in the formulation	enhanced brain availability of the drug by a factor of 7.86 and an increase (3.84-fold) in peak brain drug concentration	[130]
	the DDS has been incorporated in a thermo-responsive in situ gel made of methylcellulose to decrease mucociliary clearance	in situ gel formation with embedded hybrid NPs; enhanced relative bioavailability of the drug in the brain by a factor of about 6.4 and maximum plasma concentration reduced by a factor of 3.7	[131]
	enzyme triggered drug release in mucus, and chitosan coating enabled enhanced permeation	higher drug permeation across mucus-producing nasal epithelium cell model (RPMI 2650) compared to suspension formulation (11-fold)	[132]
	encapsulation efficiency >60% and drug release controlled by chitosan amount	sustained accumulation of the drug in the brain when administered intranasally with NPs	[124]
polymer/surfactant/cyclodextrin	thermo-responsive properties; the presence of surfactant and cyclodextrin did not alter the surface tension properties	enhancement of up to 50% in permeation compared to ropinirole solution in ex vivo permeation studies through rabbit nasal mucosa	[133]

### N2B delivery of biopharmaceuticals

Biopharmaceuticals have been described in the literature as an advanced therapeutic option for CNS diseases. One of these advanced therapeutics are monoclonal antibodies (mAbs), which are generated from B cells and are antigen-specific [134]. The advantages, including high specificity and affinity towards target antigens, make therapeutic monoclonal antibodies one of the highlights of pharmaceutical industry [135]. As a result, an increasing number of FDA-approved mAb-based therapeutics are on the market for treating CNS diseases. For example, for treating multiple sclerosis (MS), Ocrevus, based on the CD20-targeting mAb ocrelizumab, is projected to have the highest growth in sales by 2025 [136]. Moreover, aducanumab, a mAb IgG1, was approved for treating Alzheimer’s disease in 2021 [137]. Even though there are successful examples, using the full potential of mAbs for treating CNS diseases is limited. It is estimated that only 1 in 1000 antibodies can reach the brain with a concentration as low as 0.1% of the injected dose [138]. As an example, aducanumab can be mentioned. Although it has low BBB passage, it targets the brain amyloid plaque at high injection doses. However, these doses can also cause BBB disruption [139]. Researchers are working on the focused-ultrasound-mediated delivery of aducanumab by shortly opening the BBB, indicating the need for novel targeting strategies for mAbs [140]. As another example, ocrelizumab showed modest disease progress in clinical trials [141–142] although only a limited amount of the CD20 antibodies can cross the BBB. This can be attributed to their large size and degradation-prone nature [143–144].

In addition, therapy with small interfering RNA (siRNA) is also considered a powerful tool for modulating gene expression through gene silencing, and it can open new doors for disease treatments [145]. Currently, patisiran, givosiran, lumasiran, inclisiran, nedosiran, and vutrisiran are siRNA-based drugs approved by FDA for treating different diseases [146]. Besides these successful efforts, their delivery to the site of action with the highest efficiency and without toxicity or enzymatic degradation is considered a challenge [147]. Therefore, using DDSs to deliver biopharmaceuticals via N2B will provide increased stability and targeting potential.

Meredith et al. [148] and Patharapankal et al. [149] presented critical literature reviews on N2B delivery of peptides and proteins. Considering the recent advancements and publications in the field, we highlight N2B delivery of biopharmaceuticals while emphasizing mAbs, RNA delivery, and NP functionalization techniques for better targeting the brain [150].

Despite the advantages of mAbs, a limited number of studies is available with respect to N2B delivery for treating CNS diseases. The challenge of delivering these molecules and the lack of extensive research compared to other biopharmaceuticals could be attributed to their large molecular size, low stability, and high required doses, which limit their formulation options with DDSs [151]. However, the trend has changed, and more research highlighting nanotechnological approaches for delivering mAbs via the nasal route has been available [152]. For instance, Musumeci et al. worked on the absorption of mAbs neutralizing the tumor necrosis factor-related apoptosis-inducing ligand on the surfaces of polymeric NPs and NLCs for the treatment of Alzheimer’s disease. The in vivo studies in a mouse model showed that polymeric NPs and NLCs reached the brain via N2B delivery. They also showed that the adsorption of the monoclonal antibody onto the NPs led to an increase in particle size and influenced the distribution of NPs in the hippocampus, indicating another critical factor for N2B delivery, namely, the particle size of NPs [153]. In another study with mAbs, Ferreira et al. developed and optimized a system of PLGA and oligomeric chitosan for the co-delivery of alpha-cyano-4-hydroxycinnamic acid and the monoclonal antibody cetuximab to the brain for glioblastoma therapy. The cetuximab conjugation on the NP surface improved the cytotoxicity profile, and a chicken chorioallantoic membrane assay showed enhanced antiangiogenic effects for the mAbs-conjugated NPs. The authors showed that mucoadhesive NPs with enhanced antiangiogenic effects could be a good candidate [154]. In another study examining glioblastoma therapy via the antibody conjugation strategy, Chu et al. reported on ephrin type-A receptor 3 (EPHA3) tyrosine kinase antibody-modified PLGA NPs. The NPs were coated with N-trimethylated chitosan (TMC) to overcome nasal clearance and, thus, increase brain delivery. The TMC-coated NPs showed increased cellular uptake compared to the non-coated NPs in cell uptake studies with C6 cells. Moreover, when glioma-bearing rats were treated with the NPs, the median survival was increased by a factor of 1.37 [155].

**Figure 5:**
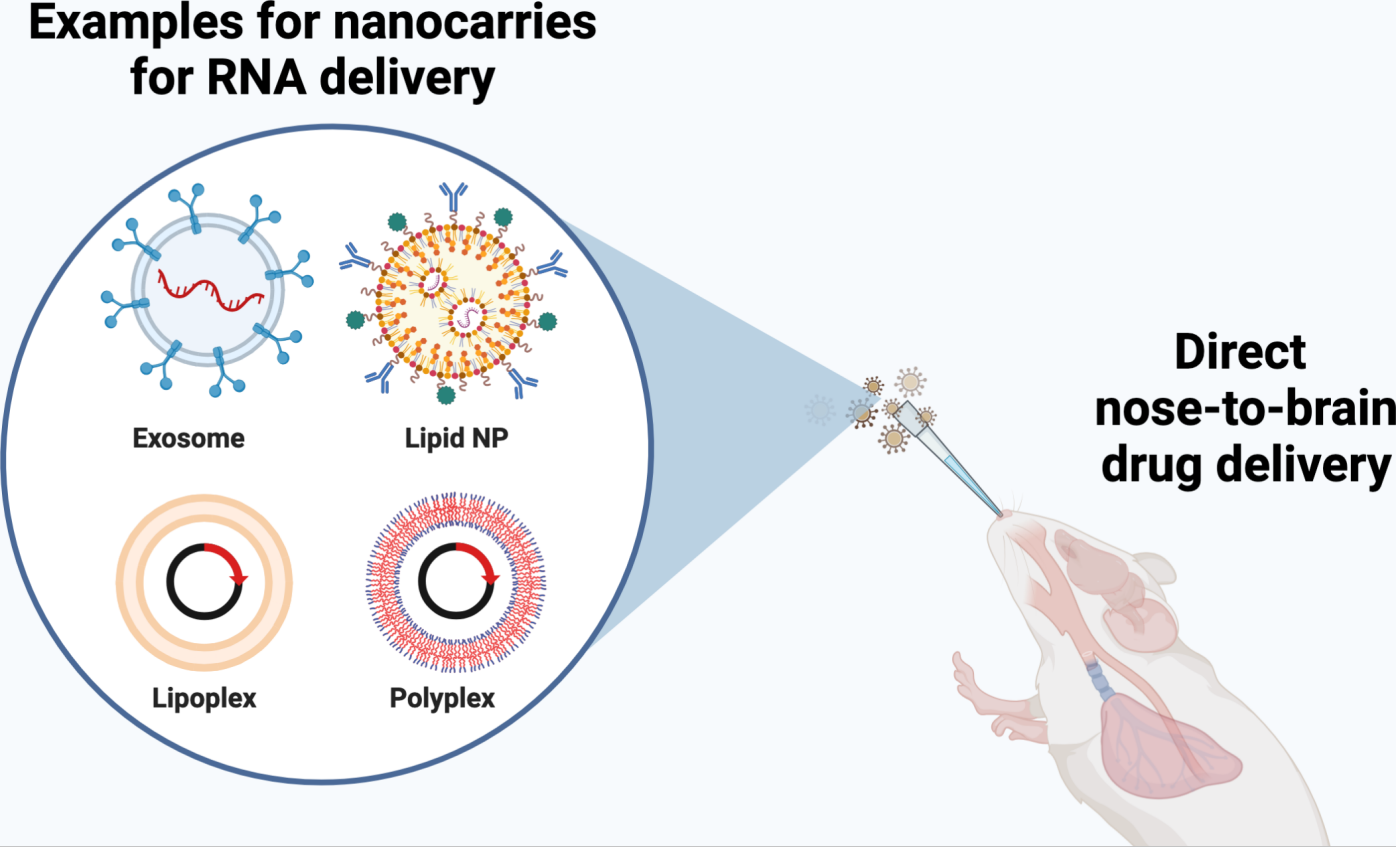
RNA delivery tools for N2B delivery. Figure was created in BioRender. Akpinar, S. (2024) https://BioRender.com/i39k654.

Regarding RNA delivery, recent literature specifically stresses the delivery of siRNA. siRNAs are regarded as an effective and attractive strategy to inhibit specific genes; therefore, there is the possibility of studying single gene function through N2B delivery [156]. In order to deliver RNA efficiently to the brain, different DDSs, such as exosomes, lipid NPs, lipoplexes, and polyplexes, were formulated, and their efficiency to successfully deliver RNA were tested in vivo (Figure 5) [157]. For example, chitosan-based nanoparticles loaded with anti-huntingtin siRNA were formulated and tested in the transgenic YAC128 mouse model of Huntington’s disease. The formulations successfully reduced the mRNA expression by at least 50% [158]. In another study, chitosan was combined with manfafodipir to allow for MRI visualization and to deliver anti-eGFP siRNA and double-stranded DNA. The formulation offered advantages over others as an effective tracker. It confirmed the accumulation of NPs in the olfactory bulbs and other regions of the brain via intranasal administration [159].

Additionally, Jeong et al. focused on another widely used complex vector, polyplexes. They took the approach one step further and combined these polyplexes formed by a cationic polymer and anionic mRNA with a photosensitizer for photochemical internalization and subsequent enhancement in delivery to the cytoplasm. They showed that laser irradiation increased the mRNA expression in the cytoplasm [160]. In addition to polyplexes, lipoplexes, another widely used vector, have also been studied for siRNA delivery. For instance, Hu et al. described the encapsulation of siRNA (specifically of c-Myc-targeting siRNA) in a core–shell lipoplex for treating glioblastoma [161].

The delivery of RNA can also be increased by functionalization. One of the most popular functionalization choices is a 29 amino-acid peptide derived from the rabies virus glycoprotein (RVG29) as it enhances brain targeting [162]. Therefore, researchers combined RVG29 and RNA delivery to further improve the expression of the RNA. For instance, Hao et al. modified RVG29 by a PEG–PLGA polymer and encapsulated microRNA(miR)-124 via double emulsion to synthesize NPs for ischemic stroke treatment. The results in in vivo studies showed that NPs entered the brain rapidly, and the modification with PEG and RVG29 improved the intranasal delivery of the NPs [163]. Li et al. also worked on RVG29 and developed core–shell lesion-recognizing NPs consisting of RVG29 peptide-modified mesenchymal stem cell-derived exosomes as the shell and a reactive oxygen species-responsive polymer loaded with siRNAs as the core for the treatment of Alzheimer’s disease. The results indicate that the formulation efficiently passed the nasal mucosa and reached the targeted brain areas [164].

## Conclusion

Despite extensive research on providing effective therapeutic strategies for CNS diseases, the treatment of these diseases remains a challenge because of the presence of the BBB. The BBB restricts the passage of large and small molecules to the brain. Intranasal drug delivery could be a noninvasive solution to these limitations. While it offers advantages due to the direct anatomical route between the brain and the nose allowing for a new path for drugs to bypass the BBB, intranasal drug delivery also has limitations. These are primarily related to the low retention time of drugs in the nasal cavity due to mucociliary clearance. Also, there might be a need for additional targeting strategies to enhance brain targeting. Providing extra stability to the drugs through their tailorable characteristics, DDSs could be used to efficiently target the brain and minimize rapid nasal clearance. They can be combined with ligands to advance the targeting to the brain and exhibit mucoadhesive properties to help the drug reach the brain.

Recent literature highlights SLNs, NLCs, liposomes, polymeric NPs, and emulsions. While lipid-based NPs are favorable because of their lipophilicity and biocompatibility, polymeric NPs offer greater control over drug release, stability, and mechanical properties [123]. Furthermore, there is much recent research on using nanoemulsions for N2B delivery for lipophilic drugs because of their high encapsulation efficiency. Moreover, hybrid NPs can exhibit enhanced properties that a single NP type does not have. Therefore, recent literature focuses on many different hybrid NPs and highlights the possibility of even more advanced and combined benefits of using NPs for intranasal drug delivery. It should also be noted that there has been a shift in the type of drug molecules studied for N2B delivery. The current research applies advanced therapeutics such as mAbs and RNA for efficient and specific targeting. In N2B delivery, DDSs provide them stability and the ability to penetrate biological barriers through their tailorable surface characteristics. Among biopharmaceuticals, siRNAs also stand out because of their selectivity for a single gene, bringing new opportunities to provide solutions to CNS diseases. Thus, N2B delivery offers significant benefits for treating CNS diseases but also suffers from limitations. Nanotechnological approaches in N2B delivery enabled enhanced properties that allow the drugs to reach the target. However, the choice of DDS and targeting strategy depends on the type of disease and the drug molecule studied. Despite the advances in N2B delivery, the current data stresses mostly preclinical studies and their outcomes. While nanotechnological applications show a large potential in N2B delivery, the transition of NP-based formulations from preclinical to clinical trials is limited. For biopharmaceutics, no such trials have been started yet. Only APH-1105, an investigational nanomaterial-based small-molecule drug product in the form of sterile, pyrogen-free lyophilized powder is investigated for the intranasal administration to treat Alzheimer’s disease in ongoing clinical trials [165]. However, more research is still needed regarding clinical studies and the commercialization of these strategies. Therefore, commercialization and scale-up should be the next steps in the future of N2B delivery research.

## Data Availability

Data sharing is not applicable as no new data was generated or analyzed in this study.

## References

[1] Chiel H J, Beer R D (1997). Trends Neurosci.

[2] Xu J, Ma C, Hua M, Li J, Xiang Z, Wu J (2022). Front Immunol.

[3] Chitnis T, Weiner H L (2017). J Clin Invest.

[4] Bergen D C, Silberberg D (2002). Arch Neurol (Chicago).

[5] Ding C, Wu Y, Chen X, Chen Y, Wu Z, Lin Z, Kang D, Fang W, Chen F (2022). Front Public Health.

[6] DiLuca M, Olesen J (2014). Neuron.

[7] Meyer A H, Feldsien T M, Mezler M, Untucht C, Venugopalan R, Lefebvre D R (2023). Pharmaceutics.

[8] Banks W A (2016). Nat Rev Drug Discovery.

[9] Greene C, Hanley N, Campbell M (2019). Fluids Barriers CNS.

[10] Bauer H C, Krizbai I A, Bauer H, Traweger A (2014). Front Neurosci.

[11] Kadry H, Noorani B, Cucullo L (2020). Fluids Barriers CNS.

[12] Cabezas R, Ávila M, Gonzalez J, El-Bachá R S, Báez E, García-Segura L M, Jurado Coronel J C, Capani F, Cardona-Gomez G P, Barreto G E (2014). Front Cell Neurosci.

[13] Knox E G, Aburto M R, Clarke G, Cryan J F, O’Driscoll C M (2022). Mol Psychiatry.

[14] Jung O, Thomas A, Burks S R, Dustin M L, Frank J A, Ferrer M, Stride E (2022). Trends Neurosci.

[15] Pardridge W M (2005). NeuroRx.

[16] Teleanu R I, Preda M D, Niculescu A-G, Vladâcenco O, Radu C I, Grumezescu A M, Teleanu D M (2022). Pharmaceutics.

[17] van den Broek S L, Shalgunov V, Herth M M (2022). Biomater Adv.

[18] Lee D, Minko T (2021). Pharmaceutics.

[19] Trevino J T, Quispe R C, Khan F, Novak V (2020). J Clin Trials.

[20] Huang Q, Chen Y, Zhang W, Xia X, Li H, Qin M, Gao H (2024). J Controlled Release.

[21] Formica M L, Real D A, Picchio M L, Catlin E, Donnelly R F, Paredes A J (2022). Appl Mater Today.

[22] Awad R, Avital A, Sosnik A (2023). Acta Pharm Sin B.

[23] Montegiove N, Calzoni E, Emiliani C, Cesaretti A (2022). J Funct Biomater.

[24] Costa C P, Moreira J N, Sousa Lobo J M, Silva A C (2021). Acta Pharm Sin B.

[25] Chaturvedi M, Kumar M, Pathak K (2011). J Adv Pharm Technol Res.

[26] Zhao K, Xie Y, Lin X, Xu W (2022). Int J Nanomed.

[27] Islam S U, Shehzad A, Ahmed M B, Lee Y S (2020). Molecules.

[28] Gizurarson S (1993). Adv Drug Delivery Rev.

[29] Irwin W J, Holbrook P A, Dey M J (1995). Int J Pharm.

[30] Sarkar M A (1992). Pharm Res.

[31] Wen P, Ren C (2024). Neuroprotection.

[32] Mitragotri S, Burke P A, Langer R (2014). Nat Rev Drug Discovery.

[33] Yue W, Shen J (2023). Pharmaceuticals.

[34] Baig M H, Ahmad K, Saeed M, Alharbi A M, Barreto G E, Ashraf G M, Choi I (2018). Biomed Pharmacother.

[35] Dighe S, Jog S, Momin M, Sawarkar S, Omri A (2024). Pharmaceutics.

[36] Alu A, Chen L, Lei H, Wei Y, Tian X, Wei X (2022). EBioMedicine.

[37] Ozsoy Y, Güngör S (2011). Expert Opin Drug Delivery.

[38] Kim D, Kim Y H, Kwon S (2018). Sci Rep.

[39] Arora P, Sharma S, Garg S (2002). Drug Discovery Today.

[40] Henriques P, Fortuna A, Doktorovová S (2022). Eur J Pharm Biopharm.

[41] Grassin-Delyle S, Buenestado A, Naline E, Faisy C, Blouquit-Laye S, Couderc L-J, Le Guen M, Fischler M, Devillier P (2012). Pharmacol Ther.

[42] Keller L-A, Merkel O, Popp A (2022). Drug Delivery Transl Res.

[43] Selvaraj K, Gowthamarajan K, Karri V V S R (2018). Artif Cells, Nanomed, Biotechnol.

[44] Boyuklieva R, Pilicheva B (2022). Biomedicines.

[45] Liu Q, Zhang Q, Gao H, Gao X (2019). Nanoparticle Systems for Nose-to-Brain Delivery. Brain Targeted Drug Delivery System.

[46] Wang Z, Xiong G, Tsang W C, Schätzlein A G, Uchegbu I F (2019). J Pharmacol Exp Ther.

[47] Sobiesk J L, Munakomi S (2023). Anatomy, Head and Neck, Nasal Cavity. StatPearls [Internet].

[48] Gänger S, Schindowski K (2018). Pharmaceutics.

[49] Chen C R, Kachramanoglou C, Li D, Andrews P, Choi D (2014). J Neurol Surg, Part B.

[50] Moon C, Jun Yoo S, Soo Han H, Aminoff M J, Daroff R B (2014). Smell. Encyclopedia of the Neurological Sciences.

[51] Lankford C K, Laird J G, Inamdar S M, Baker S A (2020). Front Cell Neurosci.

[52] Huart C, Rombaux P, Hummel T (2013). Molecules.

[53] Shirai T, Takase D, Yokoyama J, Nakanishi K, Uehara C, Saito N, Kato-Namba A, Yoshikawa K (2023). Sci Rep.

[54] Crespo C, Liberia T, Blasco-Ibáñez J M, Nácher J, Varea E (2019). Anat Rec.

[55] Ruysseveldt E, Martens K, Steelant B (2021). Front Allergy.

[56] Huff T, Weisbrod L J, Daly D T (2023). Neuroanatomy, Cranial Nerve 5 (Trigeminal). StatPearls [Internet].

[57] Li Y, Wang C, Zong S, Qi J, Dong X, Zhao W, Wu W, Fu Q, Lu Y, Chen Z (2019). J Biomed Nanotechnol.

[58] Illum L (2004). J Pharm Pharmacol.

[59] Maaz A, Blagbrough I S, De Bank P A (2021). Pharmaceutics.

[60] Hong S-S, Oh K T, Choi H-G, Lim S-J (2019). Pharmaceutics.

[61] Tashima T (2020). Molecules.

[62] Erdő F, Bors L A, Farkas D, Bajza Á, Gizurarson S (2018). Brain Res Bull.

[63] Gandhi S, Shastri D H, Shah J, Nair A B, Jacob S (2024). Pharmaceutics.

[64] Jo D H, Kim J H, Lee T G, Kim J H (2015). Nanomedicine (N Y, NY, U S).

[65] Liu Y, Tan J, Thomas A, Ou-Yang D, Muzykantov V R (2012). Ther Delivery.

[66] Handa M, Singh A, Bisht D, Kesharwani P, Shukla R (2022). Health Sci Rev.

[67] Wong C Y J, Baldelli A, Gholizadeh H, Oguzlu H, Guo Y, Xin Ong H, Rodriguez A P, Singhera G, Thamboo A, Singh A (2023). Eur J Pharm Biopharm.

[68] Koo J, Lim C, Oh K T (2024). Int J Nanomed.

[69] Rejman J, Oberle V, Zuhorn I S, Hoekstra D (2004). Biochem J.

[70] Rassu G, Soddu E, Cossu M, Gavini E, Giunchedi P, Dalpiaz A (2016). J Drug Delivery Sci Technol.

[71] Patel T, Zhou J, Piepmeier J M, Saltzman W M (2012). Adv Drug Delivery Rev.

[72] Zielińska A, Carreiró F, Oliveira A M, Neves A, Pires B, Venkatesh D N, Durazzo A, Lucarini M, Eder P, Silva A M (2020). Molecules.

[73] Ding S, Anton N, Vandamme T F, Serra C A (2016). Expert Opin Drug Delivery.

[74] Rietscher R, Thum C, Lehr C-M, Schneider M (2015). Pharm Res.

[75] Schiller S, Hanefeld A, Schneider M, Lehr C-M (2015). Pharm Res.

[76] Valencia P M, Farokhzad O C, Karnik R, Langer R (2012). Nat Nanotechnol.

[77] Ojeda-Hernández D D, Canales-Aguirre A A, Matias-Guiu J, Gomez-Pinedo U, Mateos-Díaz J C (2020). Front Bioeng Biotechnol.

[78] Wang Y, Qin B, Xia G, Choi S H (2021). AAPS J.

[79] Gabold B, Adams F, Brameyer S, Jung K, Ried C L, Merdan T, Merkel O M (2023). Drug Delivery Transl Res.

[80] Chatzitaki A-T, Jesus S, Karavasili C, Andreadis D, Fatouros D G, Borges O (2020). Int J Pharm.

[81] Spindler L M, Feuerhake A, Ladel S, Günday C, Flamm J, Günday-Türeli N, Türeli E, Tovar G E M, Schindowski K, Gruber-Traub C (2021). Front Pharmacol.

[82] Haasbroek-Pheiffer A, Van Niekerk S, Van der Kooy F, Cloete T, Steenekamp J, Hamman J (2023). Biopharm Drug Dispos.

[83] Albarki M A, Donovan M D (2020). AAPS PharmSciTech.

[84] Vachhani S, Kleinstreuer C (2021). Aerosol Sci Technol.

[85] Smith J, Wood E, Dornish M (2004). Pharm Res.

[86] Kali G, Özkahraman B, Laffleur F, Knoll P, Wibel R, Zöller K, Bernkop-Schnürch A (2023). Biomacromolecules.

[87] Sharma A, Sharma U S (1997). Int J Pharm.

[88] Pattni B S, Chupin V V, Torchilin V P (2015). Chem Rev.

[89] Sercombe L, Veerati T, Moheimani F, Wu S Y, Sood A K, Hua S (2015). Front Pharmacol.

[90] Liu G, Hou S, Tong P, Li J (2022). Crit Rev Anal Chem.

[91] Immordino M L, Dosio F, Cattel L (2006). Int J Nanomed.

[92] Nsairat H, Khater D, Sayed U, Odeh F, Al Bawab A, Alshaer W (2022). Heliyon.

[93] Giordani S, Marassi V, Zattoni A, Roda B, Reschiglian P (2023). J Pharm Biomed Anal.

[94] Vieira D B, Gamarra L F (2016). Int J Nanomed.

[95] Juhairiyah F, de Lange E C M (2021). AAPS J.

[96] Dhaliwal H K, Fan Y, Kim J, Amiji M M (2020). Mol Pharmaceutics.

[97] Salade L, Wauthoz N, Deleu M, Vermeersch M, De Vriese C, Amighi K, Goole J (2017). Int J Nanomed.

[98] Salade L, Wauthoz N, Vermeersch M, Amighi K, Goole J (2018). Eur J Pharm Biopharm.

[99] Ansari M S, Ali A, Rashid M A, Alhamhoom Y, Sultana N, Waheed A, Alam M S, Aqil M, Sultana Y (2024). J Drug Delivery Sci Technol.

[100] Ghasemiyeh P, Mohammadi-Samani S (2018). Res Pharm Sci.

[101] Duan Y, Dhar A, Patel C, Khimani M, Neogi S, Sharma P, Kumar N S, Vekariya R L (2020). RSC Adv.

[102] Weber S, Zimmer A, Pardeike J (2014). Eur J Pharm Biopharm.

[103] Viegas C, Patrício A B, Prata J M, Nadhman A, Chintamaneni P K, Fonte P (2023). Pharmaceutics.

[104] Neves A R, Queiroz J F, Weksler B, Romero I A, Couraud P-O, Reis S (2015). Nanotechnology.

[105] Correia A C, Monteiro A R, Silva R, Moreira J N, Sousa Lobo J M, Silva A C (2022). Adv Drug Delivery Rev.

[106] Prajapati J B, Patel G C (2021). J Drug Delivery Sci Technol.

[107] Zafar A, Awad Alsaidan O, Alruwaili N K, Sarim Imam S, Yasir M, Saad Alharbi K, Singh L, Muqtader Ahmed M (2022). Int J Pharm.

[108] Yasir M, Zafar A, Noorulla K M, Tura A J, Sara U V S, Panjwani D, Khalid M, Haji M J, Gobena W G, Gebissa T (2022). J Drug Delivery Sci Technol.

[109] Patel H P, Gandhi P A, Chaudhari P S, Desai B V, Desai D T, Dedhiya P P, Maulvi F A, Vyas B A (2021). J Drug Delivery Sci Technol.

[110] Hou X, Sheng J J (2023). Geoenergy Sci Eng.

[111] Shaker D S, Ishak R A H, Ghoneim A, Elhuoni M A (2019). Sci Pharm.

[112] Gaba B, Khan T, Haider M F, Alam T, Baboota S, Parvez S, Ali J (2019). BioMed Res Int.

[113] Choudhury H, Zakaria N F B, Tilang P A B, Tzeyung A S, Pandey M, Chatterjee B, Alhakamy N A, Bhattamishra S K, Kesharwani P, Gorain B (2019). J Drug Delivery Sci Technol.

[114] Gugliandolo A, Bramanti P, Mazzon E (2017). Int J Mol Sci.

[115] Dogra A, Narang R S, Kaur T, Narang J K (2024). AAPS PharmSciTech.

[116] Ahmed B, Rizwanullah M, Mir S R, Akhtar M S, Amin S (2022). Biomed Mater.

[117] Beach M A, Nayanathara U, Gao Y, Zhang C, Xiong Y, Wang Y, Such G K (2024). Chem Rev.

[118] Madkhali O A (2022). Molecules.

[119] Liong M, Lu J, Kovochich M, Xia T, Ruehm S G, Nel A E, Tamanoi F, Zink J I (2008). ACS Nano.

[120] Zhang X, Wang M, Liu Z, Wang Y, Chen L, Guo J, Zhang W, Zhang Y, Yu C, Bie T (2023). Front Drug Delivery.

[121] Ma D, Mohapatra S, Nguyen T A, Nguyen-Tri P (2019). Hybrid Nanoparticles: An Introduction. Noble Metal-Metal Oxide Hybrid Nanoparticles.

[122] Gajbhiye K R, Salve R, Narwade M, Sheikh A, Kesharwani P, Gajbhiye V (2023). Mol Cancer.

[123] Parveen S, Gupta P, Kumar S, Banerjee M (2023). Med Drug Discovery.

[124] Yousfan A, Rubio N, Hakim Natouf A, Daher A, Al-Kafry N, Venner K, Kafa H (2020). RSC Adv.

[125] Subhash Hinge N, Kathuria H, Monohar Pandey M (2023). Eur J Pharm Biopharm.

[126] Raman S, Khan A A, Mahmood S (2022). J Drug Delivery Sci Technol.

[127] Dali P, Shende P (2022). AAPS PharmSciTech.

[128] Sukumar U K, Bose R J C, Malhotra M, Babikir H A, Afjei R, Robinson E, Zeng Y, Chang E, Habte F, Sinclair R (2019). Biomaterials.

[129] Nanaki S G, Spyrou K, Bekiari C, Veneti P, Baroud T N, Karouta N, Grivas I, Papadopoulos G C, Gournis D, Bikiaris D N (2020). Pharmaceutics.

[130] Saha P, Singh P, Kathuria H, Chitkara D, Pandey M M (2023). Pharmaceutics.

[131] Uppuluri C T, Ravi P R, Dalvi A V (2021). Neuropharmacology.

[132] Clementino A R, Marchi C, Pozzoli M, Bernini F, Zimetti F, Sonvico F (2021). Front Pharmacol.

[133] Saitani E-M, Pippa N, Perinelli D R, Forys A, Papakyriakopoulou P, Lagopati N, Bonacucina G, Trzebicka B, Gazouli M, Pispas S (2024). Int J Mol Sci.

[134] Lu R-M, Hwang Y-C, Liu I-J, Lee C-C, Tsai H-Z, Li H-J, Wu H-C (2020). J Biomed Sci (London, U K).

[135] Tsumoto K, Isozaki Y, Yagami H, Tomita M (2019). Immunotherapy.

[136] Biopharma Dealmakers (2020). Biopharm Deal.

[137] Wojtunik-Kulesza K, Rudkowska M, Orzeł-Sajdłowska A (2023). Int J Mol Sci.

[138] Julku U, Xiong M, Wik E, Roshanbin S, Sehlin D, Syvänen S (2022). Fluids Barriers CNS.

[139] Pardridge W M (2020). Pharmaceuticals.

[140] Rezai A R, D’Haese P-F, Finomore V, Carpenter J, Ranjan M, Wilhelmsen K, Mehta R I, Wang P, Najib U, Vieira Ligo Teixeira C (2024). N Engl J Med.

[141] Sabatino J J, Zamvil S S, Hauser S L (2019). Cold Spring Harbor Perspect Med.

[142] Gupta S, Zamvil S S (2021). Neurol Neuroimmunol Neuroinflammation.

[143] Han L, Liu C, Qi H, Zhou J, Wen J, Wu D, Xu D, Qin M, Ren J, Wang Q (2019). Adv Mater (Weinheim, Ger).

[144] Sifniotis V, Cruz E, Eroglu B, Kayser V (2019). Antibodies.

[145] Amiri A, Barreto G, Sathyapalan T, Sahebkar A (2021). Curr Neuropharmacol.

[146] Guo S, Zhang M, Huang Y (2024). Trends Mol Med.

[147] Moazzam M, Zhang M, Hussain A, Yu X, Huang J, Huang Y (2024). Mol Ther.

[148] Meredith M E, Salameh T S, Banks W A (2015). AAPS J.

[149] Patharapankal E J, Ajiboye A L, Mattern C, Trivedi V (2024). Pharmaceutics.

[150] Rhym L H, Anderson D G (2022). Med.

[151] Quinteros D A, Bermúdez J M, Ravetti S, Cid A, Allemandi D A, Palma S D, Andronescu E, Grumezescu A M (2017). Therapeutic use of monoclonal antibodies: general aspects and challenges for drug delivery. Nanostructures for Drug Delivery.

[152] Borrajo M L, Alonso M J (2022). Drug Delivery Transl Res.

[153] Musumeci T, Di Benedetto G, Carbone C, Bonaccorso A, Amato G, Lo Faro M J, Burgaletto C, Puglisi G, Bernardini R, Cantarella G (2022). Biomedicines.

[154] Ferreira N N, Granja S, Boni F I, Prezotti F G, Ferreira L M B, Cury B S F, Reis R M, Baltazar F, Gremião M P D (2020). Drug Delivery Transl Res.

[155] Chu L, Wang A, Ni L, Yan X, Song Y, Zhao M, Sun K, Mu H, Liu S, Wu Z (2018). Drug Delivery.

[156] Dana H, Chalbatani G M, Mahmoodzadeh H, Karimloo R, Rezaiean O, Moradzadeh A, Mehmandoost N, Moazzen F, Mazraeh A, Marmari V (2017). Int J Biomed Sci (Pomona, CA, U S).

[157] Mainini F, Eccles M R (2020). Molecules.

[158] Sava V, Fihurka O, Khvorova A, Sanchez-Ramos J (2020). Nanomedicine (N Y, NY, U S).

[159] Sanchez-Ramos J, Song S, Kong X, Foroutan P, Martinez G, Dominguez-Viqueria W, Mohapatra S, Mohapatra S, Haraszti R A, Khvorova A (2018). J Drug Delivery Sci Technol.

[160] Jeong H, Kim H, Kim Y A, Kim K S, Na K (2023). ACS Appl Mater Interfaces.

[161] Hu Y, Jiang K, Wang D, Yao S, Lu L, Wang H, Song J, Zhou J, Fan X, Wang Y (2022). Acta Biomater.

[162] Liu Y, Huang R, Han L, Ke W, Shao K, Ye L, Lou J, Jiang C (2009). Biomaterials.

[163] Hao R, Sun B, Yang L, Ma C, Li S (2020). Drug Delivery.

[164] Li J, Peng H, Zhang W, Li M, Wang N, Peng C, Zhang X, Li Y (2023). ACS Appl Mater Interfaces.

[165] National Library of Medicine (U.S.) (2024). Study of APH-1105 in Patients With Mild to Moderate Alzheimer’s Disease; Identifier: NCT03806478.

